# From fragmentation to federation: multi-partner OMOP implementation in Uganda enabling global real-world evidence generation

**DOI:** 10.3389/fdgth.2026.1907568

**Published:** 2026-09-17

**Authors:** Francis Kanyike, Annet Nanungi, Harriet Dickinson, Atif Adam, James Brash, Thu Do, Caroline Otike, Michael Bogart, Alex Asiimwe, Mui Van Zandt, Cissy Kityo Mutuluuza

**Affiliations:** 1 Joint Clinical Research Centre (JCRC), Kampala, Uganda; 2 Gilead Sciences, London, United Kingdom; 3 IQVIA Inc, Durham, NC, United States; 4 Gilead Sciences, Foster City, CA, United States

**Keywords:** Africa, data harmonization, federated analytics, health informatics, HIV, OMOP Common Data Model, real-world evidence, Uganda

## Abstract

**Background:**

Incorporating geographically diverse populations into real-world evidence studies is essential for generating globally applicable insights. However, many African healthcare institutions operate highly customized electronic medical record systems that lack interoperability with international data networks. This paper describes a collaborative implementation to transform legacy electronic medical record data into the OMOP Common Data Model at the Joint Clinical Research Centre (JCRC) in Uganda, enabling participation in federated real-world evidence networks.

**Methods:**

A tri-partite collaboration was established between JCRC, IQVIA, and Gilead Sciences. The project was executed in three phases: (1) data assessment and architecture design, (2) extract, transform, and load execution and custom terminology mapping, and (3) validation of the transformed data through a cohort characterization study. JCRC's Integrated Clinical Enterprise Application (ICEA) database, containing over 48,000 patient records, was assessed and transformed to OMOP CDM v5.4. Following conversion, a descriptive observational study characterized four cohorts: all patients, people with HIV (PWH), hepatitis B virus (HBV)-infected, and hepatitis C virus (HCV)-infected patients.

**Results:**

The source database comprised 168 tables and 1,894 fields with no standardized medical coding systems —100% custom terminology— requiring manual mapping. Over 100 h of collaborative mapping achieved 95% data capture for core domains. A total of 48,748unique patients were successfully mapped to the OMOP CDM. The cohort characterization demonstrated the utility of the transformed data, revealing: HIV prevalence of 38.1% (*n* = 18,588); HBV prevalence of 7.0% (*n* = 3,423); and no HCV-infected patients identified. People with HIV demonstrated high care engagement (95.9% on antiretroviral therapy, a median of 25 visits per patient over a median follow-up of 7.6 years). Key data gaps included limited documentation of non-communicable diseases and mortality. Critical lessons included the need for detailed column-level data dictionaries and structured capacity building for local OMOP sustainability.

**Conclusions:**

This collaboration demonstrates that full OMOP CDM implementation is feasible in highly customized data environments when built on a collaborative design that combines local clinical expertise with global technical support. The project successfully positioned JCRC to engage with OHDSI's federated ecosystem and provides a replicable model for other African institutions seeking to generate representative real-world evidence at scale.

## Introduction

Real-world evidence (RWE)—defined as evidence derived from analysis of routinely collected health data—has become increasingly important for informing clinical practice, regulatory decisions, and public health policy (1, 2). However, the value of RWE depends critically on the diversity and representativeness of the underlying data sources. Incorporating geographically diverse populations into RWE studies is essential for generating insights that are globally applicable and can effectively inform public health strategies and regulatory decisions (1, 2). The underrepresentation of African populations in clinical research and real-world data analyses limits the generalizability of findings and may exacerbate health inequities (3). As regulatory agencies worldwide increasingly accept RWE for decision-making, the need for standardized, interoperable health data from diverse settings has become paramount (4).

**Table 1:** Main cohort and sub-cohorts summary.

Cohort	Number of Patients	Percentage of Main Cohort
All patients (main cohort)	48,748	100%
People with HIV (PWH)	18,588	38.1%
Current or previous HBV infection	3,423	7.0%
Current or previous HCV infection	0	0%

Data fragmentation—the existence of isolated, non-interoperable data silos—remains a major barrier to multi-site research collaboration across Africa. This fragmentation limits the ability to generate robust evidence for decision-making and perpetuates the underrepresentation of African populations in global health research. Data federation—the ability to query distributed data sources without centralizing raw data—offers a solution that respects data sovereignty while enabling collaborative research.

The Observational Medical Outcomes Partnership (OMOP) Common Data Model (CDM), maintained by the Observational Health Data Sciences and Informatics (OHDSI) community, provides a standardized framework for harmonizing observational health data across disparate sources, thereby enabling federated analytics (5, 6).The OMOP CDM enables federated analytics where data remains local while analytical code is shared, preserving data sovereignty while enabling collaborative research (7). This model is particularly attractive for African institutions seeking to contribute to global evidence generation while maintaining control over their data (8).

The research question guiding this study was: *Can a legacy electronic medical record (EMR) system with 100% custom terminology be successfully transformed to the OMOP CDM in a resource-limited setting, and can the resulting standardized data support meaningful cohort characterization and real-world evidence generation?*

The primary objective of this study was to describe and evaluate the development and implementation of an extract, transform, load (ETL) pipeline to standardize JCRC's EMR data into the OMOP CDM. The secondary objective was to validate the utility of the transformed data through a cohort characterization study demonstrating the types of analyses now possible with the standardized dataset.

The Joint Clinical Research Centre (JCRC) in Uganda is a leading institution in HIV care and research with a long-standing track record of participation in both clinical trials and observational studies. Established in 1991 as a joint venture between the Uganda Ministry of Health, Ministry of Defense, and Makerere University Medical School, JCRC pioneered the use of antiretroviral therapy (ART) for HIV treatment in sub-Saharan Africa as early as 1992 (9). Over three decades, JCRC has built a national network, conducted landmark research trials that have shaped global HIV/AIDS policies, and evolved into a regional center of excellence for research, training, and healthcare delivery.

While JCRC's electronic medical record system—the Integrated Clinical Enterprise Application (ICEA)—has supported numerous research efforts, its integration into global federated RWE infrastructures has been limited by data format and interoperability constraints. The ICEA database is a customized, home-grown system designed to address clinical care needs and programmatic reporting for HIV patients in Uganda. Like many health information systems in low- and middle-income countries (LMICs), it operates with locally developed terminologies and data structures that, while effective for clinical care, create barriers to multi-site research collaboration (10).

This challenge is not unique to Uganda. Across Africa, health data remain fragmented across disparate systems, impeding the generation of robust evidence for policy and practice. Recent initiatives across Africa have demonstrated the feasibility and value of OMOP implementation. The INSPIRE datahub has established a pan-African platform for harmonizing longitudinal population health data using OHDSI tools, demonstrating the applicability of OMOP for Health and Demographic Surveillance System (HDSS) data across multiple countries (11, 12). The LAISDAR project in Rwanda successfully implemented a federated data network of 15 hospitals using OMOP CDM, demonstrating the approach's scalability for pandemic response (13). Similarly, the Nairobi Urban HDSS COVID-19 sero-surveillance data has been successfully mapped to OMOP CDM, illustrating the model's utility for population health surveillance (14).

To further leverage the value of JCRC's clinical data within international RWE networks, a collaboration was formed between JCRC, IQVIA, and Gilead Sciences. This effort aimed not only to enable federated analytics using OHDSI tools but also to build local capacity for ongoing data standardization and research collaboration. This paper describes the implementation process, challenges encountered, results of the initial cohort characterization, and lessons learned that may inform similar initiatives across Africa.

## Methods

### Study design and setting

This study employed a retrospective observational design to describe and evaluate the process and outcomes of a data transformation project. This design is appropriate for documenting the development and implementation of a data infrastructure and for assessing the utility of the resulting standardized dataset. This was a retrospective observational study of routinely collected data from JCRC in Uganda, following transformation of source data to OMOP CDM format. The study adheres to the REporting of studies Conducted using Observational Routinely-collected health Data (RECORD) statement. The study was conducted at JCRC's main campus in Kampala, Uganda, with data originating from the ICEA database, which has been in use since 2015 and contains clinical records dating back to 1991.

### Collaboration structure and governance

The project was executed through a tri-partite collaboration, with each institution bringing complementary capabilities:

- **JCRC** contributed clinical and research expertise, local IT administration, data science leadership, and served as data owner. JCRC maintained full control over patient-level data throughout the project.
- **IQVIA** led the OMOP conversion and analytics workflow, providing ETL specialists and technical expertise in data standardization.
- **Gilead Sciences** coordinated study design, provided scientific oversight, and contributed to protocol development.

This conversion project was approved by the JCRC Research Ethics Committee (REC) and the Uganda National Council for Science and Technology (UNCST). A formal Data Sharing Agreement was established between JCRC and IQVIA prior to project commencement.

### Project phases

The project was executed in three collaborative phases:

#### Phase 1: data assessment and architecture design

IQVIA's ETL specialists worked closely with JCRC's data and clinical teams to document table relationships, field semantics, and domain alignments within the ICEA database. The source data structure was comprehensively assessed to determine suitability for OMOP CDM conversion. A Minimum Viable Product (MVP) OMOP design was proposed and tailored to support the primary research use case—characterization of HIV, HBV, and HCV patient populations—while balancing conversion complexity. This phase included establishing IT infrastructure for secure, remote access by IQVIA to the ICEA database behind JCRC's firewall.

#### Phase 2: ETL execution and custom mapping

Extract, transform, load (ETL) development involved collaborative engagement with JCRC clinical and data teams to construct custom codebooks mapping local terms to OMOP-standard vocabularies. Data were transformed to OMOP Common Data Model (CDM) version 5.4. Given that the ICEA database contained no standardized medical coding systems (e.g., ICD-10, SNOMED, LOINC), this phase required extensive manual mapping. IQVIA facilitated mapping logic and transformation implementation using OHDSI tools including WhiteRabbit for data profiling, RabbitInAHat for ETL design, and USAGI for vocabulary mapping (15). The ETL process was implemented using SQL scripts. Complete documentation, including ETL scripts and mapping specifications, was delivered to JCRC to support ongoing OMOP maintenance and future conversions.

#### Phase 3:validation through cohort characterization

Following conversion, the team developed a descriptive observational study protocol* to validate the utility of the transformed data by characterizing four cohorts derived from the OMOP-converted data:

1. All patients with at least one recorded visit in the JCRC database
2. Patients with HIV(PWH)
3. Patients with current or previous HBV infection
4. Patients with current or previous HCV infection

This characterization study served as a proof-of-concept, demonstrating that the OMOP-converted data could support meaningful analyses and generate insights comparable to those from other RWE sources. Gilead led protocol development with input from all partners; IQVIA conducted data analysis using OHDSI tools including ATLAS for cohort definition and R-based analytics packages.

### Data source: the JCRC integrated clinical enterprise application

The JCRC patient database (ICEA) is a home-grown, real-time point-of-care system customized to address clinical care needs of patients with HIV and to support programmatic reporting and evaluation. Data capture is role-based: counselors capture demographics; receptionists record visit data; clinicians capture clinical history, observations, and prescriptions, pharmacists document dispensation; and laboratory staff have dedicated interfaces for test orders and results. Death information is obtained from appointment lists, missed appointment registers, or reports from next of kin and entered by a designated officer.

### Study population and cohorts

**Main cohort**: All patients with at least one recorded visit in the JCRC database within the defined study period (1st January 2017 through 31st December 2024) representing the period after ICEA implementation through the most recent complete calendar year prior to analysis.

No exclusion criteria were applied to ensure comprehensive characterization of the dataset.

**Sub-cohorts** (non-mutually exclusive; patients with co-infections may contribute to multiple cohorts):

1. **PWH cohort**: Patients with ≥1 recording of a code matching OMOP concept identifiers for HIV (including Concept ID 439727 “Human immunodeficiency virus infection” and Concept ID 4013106 “HIV positive”) and/or evidence of HIV treatment between 1st January 2017 and most recent data.
2. **HBV cohort**: Patients with ≥1 recording of a code matching OMOP concept identifiers for HBV (including Concept ID 165806002 “Hepatitis B surface antigen detected” indicating current infection, Concept ID 40796246 “Hepatitis B virus core Antibody,” and LP38320-5 “Hepatitis B DNA viral load”). Hepatitis B surface antibody (HBsAb) and e-antigen were not used to define the cohort as they do not necessarily indicate current or previous infection.
3. **HCV cohort**: Patients with ≥1 recording of a code matching OMOP concept identifiers for HCV (including Concept ID 62944002 “Hepatitis C virus”).

### Variables and outcomes

For each cohort, the following domains were characterized:

**Demographics:** Age (median, IQR, missingness, categories <18 and ≥65 years); assigned sex at birth.
**Clinical characteristics**: Tuberculosis status; HIV/AIDS status; viral hepatitis status; pregnancy status; selected comorbidities (hypertension, diabetes, COPD, heart disease, fungal infections, chronic renal disease, depression, anxiety).
**Clinical measurements**: Weight (kg) with assessment of missingness and plausibility; height and BMI; CD4 count and viral load (for PWH).
**Healthcare utilization**: Visit types; number of visits (median, IQR); follow-up duration (median days, IQR); active status (visit within 365 days).
**Treatment patterns**: ART prescription; HBV treatment; TB treatment.
**Outcomes**: Recorded death**.**
**Data quality metrics**: Completeness (missingness rates); plausibility (out-of-range values).

### Data management and federated analytics

The project employed a federated network model to ensure data security and sovereignty. Patient-level data, including source identifiers retained in source_value fields, remained within JCRC's institutional environment throughout the conversion process and were never shared externally. Analytical packages were developed by IQVIA and shared with JCRC's data team, who executed the packages locally against the OMOP-converted database. The analytical outputs shared externally consisted solely of aggregated summary statistics with cell size suppression (≥5), ensuring no individually identifiable information was disclosed. These outputs were reviewed by JCRC and, if acceptable, shared with IQVIA for population of result tables and subsequent sharing with Gilead. This approach aligns with best practices for multi-institutional data sharing where individual-level data cannot be centralized due to governance or consent restrictions (16).

### Statistical analysis

This was a descriptive study; no inferential analyses were conducted. Categorical variables were summarized using counts and percentages. Continuous variables were summarized using mean, standard deviation, median, interquartile range, minimum, and maximum where appropriate. Data quality and missingness were assessed and reported. All analyses were conducted using OHDSI analytical tools within the R statistical computing environment.

### Ethical considerations

The study protocol was reviewed and approved by the JCRC Research Ethics Committee and subsequently by the Uganda National Council for Science and Technology. As this was a secondary use analysis of de-identified data originally collected for clinical purposes, the requirement for individual informed consent was waived by the ethics committees. All patient identifiers were removed during data processing, and the analytical datasets contained no personally identifiable information. The study was conducted in accordance with Good Pharmacoepidemiology Practices (GPP) and relevant modules of the Heads of Medicines Agencies (HMA) Good Pharmacovigilance Practices (GVP).

## Results

### Data mapping and conversion outcomes

The ICEA source database presented significant challenges for OMOP conversion. Assessment revealed:

- 168 tables and 1,894 fields requiring evaluation for mapping
- No standardized medical coding systems—100% custom terminology
- Complex table relationships with limited documentation
- Single-user remote access only, constraining parallel development

Despite these challenges, over 100 person-hours of collaborative custom mapping were completed across weekly joint sessions between IQVIA ETL specialists and JCRC clinical and data teams, achieving successful transformation of approximately 95% of source records into the seven core OMOP domains (person, observation_period, visit_occurrence, condition_occurrence, drug_exposure, measurement, and observation). A total of 48,748 unique patients were successfully mapped to the OMOP CDM, representing all patients with at least one recorded visit in the database. The remaining 5% of source records were not transformed as they represented data from fields or domains that were out of scope for the Minimum Viable Product (MVP) design or were subject to data quality issues (e.g., non-standard free-text fields not mapped).

Total patient volume grew from 17,075 in 2017 to 20,036 in 2024 (17.3% increase). The proportion of HIV patients declined from 71.6% in 2017 to 67.0% in 2024, suggesting diversification beyond HIV-focused care. HBV prevalence increased more than fivefold from 2.5% in 2017 to 13.4% in 2024, likely reflecting improved screening rather than true epidemiological change. No HCV cases were documented throughout the study period, and as such, this cohort will not be described.

### Key demographic and clinical findings

**Main cohort** (*n* = 48,748): The median age was 33 years (IQR: 22–46), with 19.5% aged <18 years and 4.0% aged ≥65 years. Females comprised 53.9% of the total cohort, with higher representation (60.2%) among recently active patients. Tuberculosis positivity was documented in 34.9% of all patients but notably higher (65.9%) in active patients. Clinical measurements, comorbidities, and outcomes for the main cohort are presented in Table 2.

**Table 2:** Clinical measurements, comorbidities, and outcomes.

Variable	All Patients (*n* = 48,748)	People with HIV (*n* = 18,588)	HBV-Infected (*n* = 3,423)
**Weight measurements**			
**Weight (kg), median (IQR)**	58 (48–70)	62 (53–72)	62 (53–72)
**Missing weight, *n* (%)**	24,316 (49.9%)	6,347 (34.1%)	1,014 (29.6%)
**>1 weight measurement, *n* (%)**	16,433 (33.7%)	10,616 (57.1%)	2,069 (60.4%)
**Height and BMI**			
**Height recorded, *n* (%)**	—	5,537 (29.8%)	—
**BMI recorded, *n* (%)**	—	3,952 (21.3%)	—
**Outcomes**			
**Recorded death, *n* (%)**	328 (0.7%)	103 (0.6%)	29 (0.8%)
**Pregnancy (female patients)**			
**Any pregnancy record, *n* (%)**	339 (1.3%)^a^	1,329 (11.8%)^b^	200 (9.9%)^c^
**Selected comorbidities**			
**Hypertension, *n* (%)**	5 (<0.1%)	2 (0.01%)	—
**Diabetes, *n* (%)**	14 (0.03%)	7 (0.04%)	—
**Fungal infections, *n* (%)**	1,563 (3.2%)	1,009 (5.4%)	—

IQR, interquartile range; BMI, body mass index; PWH, people with HIV; HBV, hepatitis B virus.

a Denominator: 26,289 female patients in main cohort.

b Denominator: 11,251 female patients in PWH cohort.

c Denominator: 2,018 female patients in HBV cohort.

**People with HIV cohort** (*n* = 18,588): This cohort demonstrated high care engagement, with 95.9% receiving antiretroviral therapy (ART) and 72.2% having visits within the past year. The median follow-up duration was 7.6 years (2,779 days), with a median of 25 visits per patient. TB co-infection was 80.4%, and HBV co-infection was 15.4%—more than twice the prevalence in the general population. Female patients comprised 60.5% of the cohort. Detailed data on clinical measurements, comorbidities, and outcomes for people with HIV are presented in Table 2, while treatment patterns and healthcare utilization are summarized in Table 3.

**Table 3:** Treatment patterns and healthcare utilization.

Variable	People with HIV (*n* = 18,588)	HBV-Infected (*n* = 3,423)
**Treatment**		
**Receiving ART, *n* (%)**	17,819 (95.9%)	—
**Number of ART prescriptions, median (IQR)**	24 (7–56)	—
**Receiving HBV treatment, *n* (%)**	—	2,614 (76.4%)
**Healthcare utilization**		
**Active patients (visit within 365 days), *n* (%)**	13,427 (72.2%)	2,689 (78.6%)
**Number of visits, median (IQR)**	9 (3–26)	8 (2–25)
**Follow-up duration (days), median (IQR)** ^a^	2,779 (1,252–4,843)	2,129 (783–4,308)
**Laboratory monitoring**		
**CD4 count recorded, *n* (%)**	13,449 (72.4%)	—
**Viral load test recorded, *n* (%)**	14,261 (76.7%)	—
**Number of viral load tests, median (IQR)**	3 (1–8)	—
**Hepatitis B core antibody test, *n* (%)**	—	2,853 (83.3%)

a For patients with at least two visits.

**Abbreviations:** ART, antiretroviral therapy; IQR, interquartile range; HBV, hepatitis B virus.

**HBV-infected cohort** (*n* = 3,423): The median age was 37 years (IQR: 27–47), with females comprising 59.0%. HIV co-infection was remarkably high at 83.5%, and TB co-infection was documented in 49.3% of patients. Pregnancy was recorded in 9.9% of female patients. Median follow-up was 2.4 years (880 days) with a median of 12 visits. Clinical measurements, comorbidities, and outcomes for the HBV cohort are presented in Table 2, while treatment patterns and healthcare utilization are summarized in Table 3.

### Critical data quality findings

Several significant data quality issues were identified, assessed using the completeness and plausibility metrics defined in the methods.

**Weight measurements** showed plausibility concerns, with implausible values (mean 132.8 kg, SD 255.2) and were missing for 49.9% of patients (completeness: 50.1% captured). The extreme mean suggests probable unit inconsistencies (pounds versus kilograms) or data entry errors. JCRC uses kilograms as the standard unit for weight measurement (pounds are not used); therefore, the extreme mean suggests data entry errors rather than mixed measurement systems. This finding has informed local data quality improvement initiatives.

**Comorbidity documentation** demonstrated low completeness across all cohorts, with hypertension recorded in <5 patients, diabetes in 0.03%, and no COPD or heart disease diagnoses despite the cohort's age distribution and known cardiovascular risk in HIV patients.

**Mortality documentation** also showed low completeness across all cohorts (0.6%–0.8%), likely reflecting incomplete death reporting rather than true survival rates.

**Pregnancy records** were documented in only 1.3% of female patients overall (11.8% in HIV-positive females), indicating substantial underreporting (completeness: 1.3% of female patients had any pregnancy record).

### Key implementation insights

Several critical insights emerged from the implementation process:

**Local terminology variation** (e.g., tuberculosis algorithms, HIV staging, death recording) required continuous expert validation by JCRC clinicians. Standard mapping tables alone were insufficient; iterative review by clinical domain experts was essential to ensure fidelity**.**
**Embedded co-development** with JCRC clinicians and analysts was critical to success. Weekly collaborative sessions enabled real-time problem-solving and knowledge transfer, building local capacity for future OMOP maintenance.
**Documentation gaps** in the source database necessitated extensive reverse engineering. A detailed, column-level data dictionary was developed through this project to support all future data initiatives.
**Data quality findings** from the characterization study highlighted areas requiring attention: weight measurement inconsistencies, minimal comorbidity documentation, low mortality recording, and absence of HCV cases. These findings have informed local data quality improvement initiatives.
**Capacity building** was intentionally integrated throughout the project. Rather than delivering a “black box” conversion, the partnership structured knowledge transfer so JCRC's data team could assume ownership of OMOP maintenance and future conversions. Specific deliverables included: (1) SQL-based ETL scripts with inline comments explaining logic for each transformation; (2) custom codebooks mapping local ICEA terminology to OMOP-standard vocabularies; (3) documentation of table relationships and field semantics; and (4) a detailed column-level data dictionary covering all 1,894 source fields. These assets enable JCRC's data team to independently maintain, update, and extend the OMOP instance for future research questions without external technical support.

Weekly collaborative sessions brought together IQVIA's ETL specialists with JCRC's data and clinical teams. These sessions were not passive training but active problem-solving: IQVIA specialists explained mapping decisions in real time, JCRC analysts contributed clinical context that refined the mappings, and together they worked through complex cases such as tuberculosis algorithms, HIV staging documentation, and death recording. This model ensured that by project completion, JCRC's team understood not just what the ETL scripts did, but why each mapping decision was made—enabling them to adapt the processes independently.

The impact of this collaboration extended beyond the immediate conversion. JCRC has since strengthened its data collection practices, including implementing validation rules to address identified quality issues such as weight unit inconsistencies, and has established a formal data governance framework to support ongoing OMOP maintenance. The project also demonstrated the value of detailed documentation, leading to improved practices in source data management and a commitment to maintaining column-level metadata for future data initiatives.

Furthermore, the foundational experience from this project positioned JCRC to play a central role in organizing the inaugural OHDSI Africa Symposium in November 2025, hosted by JCRC in Kampala. This event brought together over 165 participants from across Africa and globally, demonstrating the growing regional commitment to standardized health data and the OHDSI framework.

Building on this momentum, JCRC has secured continued support to expand the OMOP initiative to include PrEP-related variables, with a focus on supporting evaluation of Lenacapavir rollout for HIV prevention in Uganda. This next phase will further strengthen local capacity for real-world evidence generation and ensure the OMOP instance remains a sustainable resource for future research.

## Discussion

This collaboration between JCRC, IQVIA, and Gilead demonstrates that full OMOP CDM implementation and cohort characterization are feasible in highly customized data environments when built on a foundation of collaborative design. The successful conversion of JCRC's legacy ICEA database—with its 168 tables, 1,894 fields, and complete absence of standardized medical coding—represents a significant achievement with implications for similar institutions across Africa seeking to participate in global RWE networks.

The primary finding of this study is that a legacy EMR system with 100% custom terminology can be successfully transformed to the OMOP CDM through a structured, collaborative process combining local clinical expertise with global technical support. The secondary finding—demonstrated through the cohort characterization—is that the transformed data can support meaningful analyses comparable to those from other RWE sources, generating clinically relevant insights while also illuminating data quality gaps that can inform local improvement efforts.

### Feasibility in resource-limited settings

Our experience aligns with and extends findings from other African OMOP implementations. The INSPIRE datahub has demonstrated the feasibility of harmonizing longitudinal population health data from multiple African HDSS sites (11, 12). The LAISDAR project in Rwanda successfully implemented OMOP CDM across 15 hospitals (13). Our project contributes the experience of a single large treatment center with a highly customized EMR, illustrating that even fragmented data environments can be transformed when local expertise is combined with technical support.

The complete absence of standardized terminologies presented challenges similar to those reported by other LMIC implementations (14). Our experience reinforces that vocabulary mapping remains the most labor-intensive aspect of OMOP conversion in settings without pre-existing coding standards, requiring over 100 person-hours of collaborative effort. However, once completed, these mappings create reusable assets that reduce the burden for future conversions.

### Clinical and data quality insights

The cohort characterization provided valuable insights into JCRC's patient population. The observed HIV prevalence of 38.1% reflects JCRC's historical focus, though the declining proportion of HIV patients suggests successful diversification into other clinical areas. The fivefold increase in HBV detection likely reflects improved screening—demonstrating the value of OMOP-converted data for monitoring programmatic interventions. The high HBV-HIV co-infection rate (83.5%) underscores the importance of integrated screening and treatment programs (17).

The complete absence of HCV cases despite known prevalence of approximately 1% in Uganda (18) raises important questions about diagnostic practices or data capture, illustrating how OMOP characterization studies can identify gaps in clinical care.

The data quality findings-implausible weight values, minimal comorbidity documentation, low mortality recording-are consistent with challenges reported in other African EMRs (19–21). The OMOP conversion process itself illuminated these gaps, demonstrating its value for data quality improvement beyond research applications. The JCRC data team is now implementing validation rules to address identified issues.

### Federated analytics and data sovereignty

The federated network model employed-where patient-level data remained at JCRC behind institutional firewalls, with only aggregated results shared-represents a paradigm particularly suited to African contexts where data sovereignty concerns are paramount (22). This approach aligns with the African Union's data policy frameworks and enables participation in global research without relinquishing local control (23). Our implementation confirms that OHDSI tools are well-suited to this model (16). This approach addresses a critical barrier to LMIC participation in global research networks—the “helicopter research” (also referred to as “parachute research”) phenomenon where external partners extract data without building local capacity (24).

### Capacity building and sustainability

The deliberate integration of capacity building throughout the project—through co-development, documentation, and training—enabled JCRC to assume full ownership and leadership of the OMOP instance post-conversion, ensuring local control over data and analytics.

### Lessons learned and recommendations

Several lessons may inform similar initiatives:

- Invest in detailed source data documentation upfront. The initial high-level data dictionary was insufficient; column-level documentation with clinical experts was essential.
- Plan for iterative vocabulary mapping. Manual review by clinical experts was essential for accurate mapping of local terminology.
- Build quality assessment into the conversion process. Early identification of data quality issues enabled corrective action and informed interpretation.
- Document everything for sustainability. Comprehensive documentation enabled knowledge transfer and positions JCRC for independent future work.
- Engage clinical experts throughout. Clinician involvement was critical for interpreting local terminology and ensuring clinical relevance.
- Consider broader stakeholder engagement. Future efforts would benefit from engagement with national bodies such as Ministries of Health to support uniform interpretation of terminologies and reference values, strengthening national interoperability

### Limitations

This study has several limitations. First, the characterization is descriptive only; no inferential comparisons were conducted. Second, the real-world data source has inherent limitations including potential misclassification, missing data, and incomplete outcome ascertainment, particularly for mortality. Third, some patients may have participated in clinical trials, which could affect observability and treatment patterns. Fourth, the absence of HCV cases precluded characterization of that cohort. Fifth, JCRC is a specialized research and treatment center, and its data systems and patient population may differ from those of other Ugandan or African health facilities. As such, while the implementation approach is replicable, each institution and EMR system will face unique contextual challenges. The lessons learned should be adapted to local contexts rather than applied directly.

### Future directions

JCRC is now positioned for improved data capture and broader observational research opportunities, including participation in OHDSI network studies, contributing African patient data to global RWE generation. Planned studies include characterizing pregnancy outcomes in HIV-positive women, evaluating long-term ART safety, and examining HBV treatment patterns. Building on this foundation, ongoing work is expanding the OMOP initiative to include PrEP-related variables, with a focus on supporting evaluation of Lenacapavir rollout for HIV prevention in Uganda. This next phase further strengthens local capacity for real-world evidence generation and ensures the OMOP instance remains a sustainable resource for future research. Future technical work includes integrating additional data sources and deploying the OHDSI stack for local researchers.

## Conclusions

This collaboration between JCRC, IQVIA, and Gilead demonstrates that full OMOP CDM implementation and characterization are feasible in highly customized data environments when built on a foundation of collaborative design. The project illustrates how local clinical and data expertise, jointly applied with global technical and scientific support, can rapidly convert unstructured health data into standardized, reusable datasets aligned with international standards.

The successful conversion of JCRC's ICEA database—from 168 tables with 100% custom terminology to a standardized OMOP instance enabling federated analytics—provides a replicable model for other African institutions seeking to generate representative real-world evidence at scale. Beyond the technical achievement, the project has built sustainable local capacity through deliberate knowledge transfer, equipping JCRC's team with ETL scripts, mapping documentation, and a detailed data dictionary that enable independent maintenance and future expansions.

This foundation has positioned JCRC to lead regional initiatives, including hosting the inaugural OHDSI Africa Symposium in 2025, and to advance ongoing work expanding the OMOP framework to PrEP-related variables in support of Lenacapavir evaluation. These developments underscore the project's lasting impact on local research capacity and data infrastructure.

As regulatory and public health decision-making increasingly relies on real-world evidence, ensuring that African populations are represented in these analyses is essential for equitable and globally applicable knowledge. Multi-partner collaborations that combine local expertise with technical resources, structured for capacity building and sustainability, offer a path toward achieving this representation while respecting data sovereignty and strengthening local institutions.

## Data Availability

The original contributions presented in the study are included in the article/Supplementary Material, further inquiries can be directed to the corresponding author.
